# Computer-aided screening of aspiration risks in dysphagia with wearable technology: a Systematic Review and meta-analysis on test accuracy

**DOI:** 10.3389/fbioe.2023.1205009

**Published:** 2023-06-27

**Authors:** Derek Ka-Hei Lai, Ethan Shiu-Wang Cheng, Hyo-Jung Lim, Bryan Pak-Hei So, Wing-Kai Lam, Daphne Sze Ki Cheung, Duo Wai-Chi Wong, James Chung-Wai Cheung

**Affiliations:** ^1^ Department of Biomedical Engineering, Faculty of Engineering, The Hong Kong Polytechnic University, Hong Kong, China; ^2^ Department of Electronic and Information Engineering, Faculty of Engineering, The Hong Kong Polytechnic University, Hong Kong, China; ^3^ Sports Information and External Affairs Centre, Hong Kong Sports Institute Ltd, Hong Kong, China; ^4^ School of Nursing, The Hong Kong Polytechnic University, Hong Kong, China; ^5^ Research Institute of Smart Ageing, The Hong Kong Polytechnic University, Hong Kong, China

**Keywords:** aspiration pneumonia, dementia, computer-aided diagnosis, gerontechnology, deep learning, machine learning

## Abstract

Aspiration caused by dysphagia is a prevalent problem that causes serious health consequences and even death. Traditional diagnostic instruments could induce pain, discomfort, nausea, and radiation exposure. The emergence of wearable technology with computer-aided screening might facilitate continuous or frequent assessments to prompt early and effective management. The objectives of this review are to summarize these systems to identify aspiration risks in dysphagic individuals and inquire about their accuracy. Two authors independently searched electronic databases, including CINAHL, Embase, IEEE Xplore^®^ Digital Library, PubMed, Scopus, and Web of Science (PROSPERO reference number: CRD42023408960). The risk of bias and applicability were assessed using QUADAS-2. Nine (n = 9) articles applied accelerometers and/or acoustic devices to identify aspiration risks in patients with neurodegenerative problems (e.g., dementia, Alzheimer’s disease), neurogenic problems (e.g., stroke, brain injury), in addition to some children with congenital abnormalities, using videofluoroscopic swallowing study (VFSS) or fiberoptic endoscopic evaluation of swallowing (FEES) as the reference standard. All studies employed a traditional machine learning approach with a feature extraction process. Support vector machine (SVM) was the most famous machine learning model used. A meta-analysis was conducted to evaluate the classification accuracy and identify risky swallows. Nevertheless, we decided not to conclude the meta-analysis findings (pooled diagnostic odds ratio: 21.5, 95% CI, 2.7–173.6) because studies had unique methodological characteristics and major differences in the set of parameters/thresholds, in addition to the substantial heterogeneity and variations, with sensitivity levels ranging from 21.7% to 90.0% between studies. Small sample sizes could be a critical problem in existing studies (median = 34.5, range 18–449), especially for machine learning models. Only two out of the nine studies had an optimized model with sensitivity over 90%. There is a need to enlarge the sample size for better generalizability and optimize signal processing, segmentation, feature extraction, classifiers, and their combinations to improve the assessment performance.

**Systematic Review Registration:** (https://www.crd.york.ac.uk/prospero/), identifier (CRD42023408960).

## 1 Introduction

Aspiration occurs when oropharyngeal contents, such as food, liquid, saliva, or secretion, are accidentally misdirected into the larynx, lower respiratory tract, or lung (Ebihara et al., 2016), which may result in aspiration pneumonia if infection or inflammation develops. Aspiration could be life-threatening when the airway is blocked (i.e., asphyxiation), and aspiration pneumonia was ranked as the third leading cause of injury deaths in older people (Kramarow et al., 2014). A study on 784 patients reported that 65.2% demonstrated pharyngeal residue-related dysphagia (Seo et al., 2021). Aspiration pneumonia resulted in more than 58,000 annual deaths in the United States, with an age-adjusted mortality rate of 21.85 per 100,000 people (Gupte et al., 2022). Another study found that the median hospitalization charge for aspiration pneumonia was US$30,526 (Wu et al., 2017). Patients who suffered from aspiration reported fear, depression, and frustration that they might aspirate again, which affected their mental health and quality of life (Martino et al., 2009).

Dysphagia or deglutition disorder (i.e., difficulty swallowing) is the main cause of aspiration (Morley, 2015). Individuals who suffered from dysphagia were about nine times more likely to develop aspiration pneumonia (van der Maarel-Wierink et al., 2011). Likewise, dysphagia was present in 92% of pneumonia patients (Almirall et al., 2013). While the prevailing dysphagic aspiration has imposed a heavy burden on the healthcare and hospitalization systems (Allen et al., 2020; Lesa et al., 2021), early diagnosis and screening of dysphagia and aspiration risks are essential to facilitate effective management and reduce subsequent risks of pulmonary complications (Hines et al., 2016; Wirth et al., 2016). Nevertheless, aspiration resulting from dysphagia is often referred to as “silent aspiration”. Some patients may remain clinically asymptomatic, without presenting coughing or choking signs, and self-report swallowing difficulties (Wakasugi et al., 2008; Miller et al., 2009; Suiter et al., 2020). Confirmation of apparent aspiration cannot be obtained clinically (Teramoto, 2022). Bedside assessment frequently misses dysphagia patients with silent aspiration (Wakasugi et al., 2008). Meanwhile, the gold standards for assessing dysphagic aspiration are the videofluoroscopic swallowing study (VFSS) and the fiber-optic endoscopic evaluation of swallowing (FEES). Nevertheless, both fluoroscopy and endoscopy induce pain, discomfort, nausea, and radiation exposure, especially in children (Ingleby et al., 2021), which are not feasible to facilitate continuous or frequent assessments. There standards also require high costs and professionals to operate (Lancaster, 2015).

As dysphagia or neurodegenerative problems deteriorate gradually and aspiration could occur unexpectedly (Lim et al., 2023), it is necessary to develop accessible and reliable instrumental screening tools that enable continuous or frequent assessments of aspiration risks. Wearable technology with computer-aided diagnosis/screening might be a potential alternative to bedside questionnaires and instrumental diagnostic instruments (such as VFSS and FEES) (So et al., 2023). Accelerometers are among the most common sensors used in wearable technology, and have been used to evaluate levels of physical (Karas et al., 2022), ambulatory (Steins et al., 2014), and behavioral information (Cheung et al., 2022). In the case of swallowing, accelerometers can trace the biomotion of the laryngeal region that manifests swallowing abnormalities and thus aspiration risks (So et al., 2023). In the same vein, soft sensors with flexible electronics or artificial skin could serve the same purpose (Chen J. et al., 2021; Chen et al., 2021b; Gao et al., 2021). On the other hand, aspirated patients may demonstrate a wet voice (Warms and Richards, 2000) and attenuated breathing and swallowing sounds (Shaw et al., 2004; Kang et al., 2017), in which acoustic features could be recognized by microphones.

Computer-aided screening, using machine learning and deep learning, can enhance the assessment of swallowing functions and, hence, dysphagia or aspiration in older adults. Park et al. (2023) attempted to predict aspiration by applying machine learning models to a bedside screening questionnaire (GUSS test). They attained an area under the receiver operating characteristics curve (AUC) of 0.81. Through the examination of videofluoroscopic hyoid motions, Lee et al. (2016) detected swallowing impairment with strong discriminative power (AUC = 0.93) using the support vector machine (SVM). In addition, Roldan-Vasco et al. (2021) categorized swallowing dysfunctions by speech variations using the random forest and obtained a 91.0% sensitivity. Nevertheless, current wearable technology was deemed insufficiently reliable to recognize swallowing and non-swallowing, which hindered real-world applications (So et al., 2023).

To this end, our review question is: how were wearable technologies with computer-aided screening techniques were utilized to identify aspiration risks in dysphagia, and how accurate were these techniques or systems, in general? The objective of this review is to summarize the evidence on the testing techniques, protocols, and accuracy performances for the assessment of aspiration risks. The Preferred Reporting Items for Systematic Review and Meta-Analyses (PRISMA-DTA) extension for diagnostic test accuracy was adopted to frame the reporting of this review. The review was registered in PROSPERO (reference number: CRD42023408960).

## 2 Materials and methods

### 2.1 Eligibility criteria

The search strategy was designed with reference to the PIRO tool (i.e., population, index test, reference test, and outcomes), and the study design of the eligible articles shall focus on the assessment of test accuracy (i.e., non-experimental cross-sectional study) with prediction models (e.g., statistical modeling, machine learning, and deep learning). For the population, we targeted individuals with dysphagia and its association with aspirations or the risks of aspirations. For the index test, our search terms were categorized into those related to screening instruments and classifiers. We did not consider search terms for reference tests because it would misdirect the search results from screening tools to diagnostic tools. Lastly, outcome variables shall be related to test or classification performance (either per-individual or per-sample).

### 2.2 Information sources

Two independent authors (DK-HL and ES-WC) searched the literature in March 2023 from electronic databases, including CINAHL (Cumulated Index to Nursing and Allied Health Literature) via EBSCOhost (default field), Embase (title, abstract, keywords) via OVID, IEEE Xplore^®^ Digital Library (metadata), PubMed (title/abstract), Scopus (title, abstract keywords), and Clarivate Web of Science (topic). There was no constraint on the year of publication, but it was limited to those in English.

### 2.3 Search strategy

The search terms were determined by snowballing literature from simple pilot searches using keywords or free-text words from the identified concept (i.e., the PIRO tool) (Aromataris and Riitano, 2014). The categories of search terms for dysphagia were “dysphagia”, “swallowing disorder”, “swallowing disorders”, “deglutition disorder”, and “deglutition disorders”. Aspiration-related terms were “aspiration”, “aspirated”, “choke”, “choking”, “inhale”, and “inhaled”. The search terms for instruments were “accelero*”, “acoustic”, “vibration”, “vibrate”, “vibratory”, “vibrated”, “sound” “stress”, “strain”, “stretch”, “stretchable”, “stretching”, “bend”, “track”, “tracking”, “sonic”, “pressure”, “resist*”, “piezo*”, “capacity*”, “film”, “nano*”, “carbon*”, “graphene”, “biomaterial”, “biosensor, “biosensors”, “sensor”, “sensors”, “artificial skin”, “soft electronics”, “flexible electronics”, “ultrasound”, “MMG”, “mechanomyography”, “microphone”. The search terms for classifiers were “machine learning”, “deep learning”, “regression”, “Bayesian”, supervised learning”, “unsupervised learning”, “reinforcement learning”, “reinforced learning”, “artificial intelligence”, “classify”, “classified”, “classification”, “cluster”, “clustering”, “SVM”, “support vector machine”, “random forest”, “decision tree”, “decision trees”, “neural network”, “neural networks”, “gradient boosting”, “XGBoost”, “AdaBoost”, “perceptron”, “transformer”, “CNN”, “RNN”, “ANN”, “KNN”, and “MLP”. The search terms were combined by an OR operation within the category and an AND operation between the categories.

### 2.4 Selection process

Inclusion criteria for the search and screen included: 1) original research articles; 2) published in English; 3) published as journal articles (in-press inclusive), preprints, or conference full papers; 4) involved individuals with dysphagia, regardless of the cause of dysphagia; 5) involved instrumental screening, e.g., accelerometers, microphones, and strain sensors; 6) binary classification of aspirated and non-aspirated individuals; or swallows with high and low risks of aspiration, airway invasion, or airway entry; 7) classifiers using statistical modeling, machine learning, or deep learning; 8) reported accuracy-related test performance measures of model predictions, such as sensitivity and specificity.

Exclusion criteria included 1) published as reviews, perspective articles, commentary, conference abstracts, book sections/chapters, or patents; 2) classification of dysphagia and non-dysphagia without accounting for aspiration; 3) index tests targeted on diagnostic equipment or invasive instruments, such as videofluoroscopic swallowing study (VFSS), fiberoptic endoscopic evaluation of swallowing (FEES), and manometry; 4) non-instrumental classification, such as observation and palpation, bedside questionnaires, and data analytics based on patients’ history and clinical records.

### 2.5 Data collection and extraction

In the screening stage, the first author (DK-HL) screened the search results by their titles, abstracts, and keywords. Thereafter, the same author assessed the eligibility of the screened results by reading articles in full text. The screened and excluded records were checked by the third author (H-YL). Any disagreement was resolved by seeking consensus with the corresponding authors. The data related to PIRO were summarized and tabulated into participant information, index test (instrument configuration and testing protocol, feature extraction, modeling), reference test, and outcome metrics and testing performance. In cases of multiple populations and tests, they would all be presented in the data synthesis tables.

### 2.6 Methodological quality assessment

The Quality Assessment of Diagnostic Accuracy Studies-2 (QUADAS-2) was used to assess the applicability and risk of bias of the reviewed articles (Whiting et al., 2011). The tool consisted of seven items and was structured into four domains, including patient selection, index test, reference standard, and flow and timing. Table 1 summarizes the domain and signaling questions for assessing the quality. The graphical presentation of QUADAS-2 results was generated using Review Manager (RevMan) version 5.4 (The Cochrane Collaboration, 2020).

**TABLE 1:** Domains and signaling questions in QUADAS-2 (Whiting et al., 2011).

Domain	Assessment	Signaling questions
Patient selection	Risk of bias	Was a consecutive or random sample of patients enrolled?
		Was a case-control design avoided?
		Did the study avoid inappropriate exclusions?
	Applicability concerns	Are there concerns that the included patients and setting do not match the review question?
Index test	Risk of bias	Were the index test results interpreted without knowledge of the results of the reference standard?
		If a threshold was used, was it pre-specified?
	Applicability concerns	Are there concerns that the index test, its conduct, or interpretation differ from the review question?
Reference test	Risk of bias	Is the reference standard likely to correctly classify the target condition?
		Were the reference standard results interpreted without knowledge of the results of the index test?
	Applicability concerns	Are there concerns that the target condition as defined by the reference standard does not match the question?
Flow and timing	Risk of bias	Was there an appropriate interval between index test and reference standard?
		Did all patients/samples receive the same reference standard?
		Were all patients/samples included in the analysis?

### 2.7 Meta-analysis

We considered sensitivity and specificity as the principle diagnostic accuracy measures, though we also listed out the PPV (positive predictive value), NPV (negative predictive value), AUC, and other outcomes in the table. Sensitivity and PPV were also known as recall and precision, respectively. The number of true positives, true negatives, false positives, and false negatives (i.e., 2 × 2 contingency table, or the confusion matrix) were estimated by the available information of sensitivity, specificity, and the approximated size of the testing dataset from the cross-validation ratio from the papers. We only considered one outcome for each study in the meta-analysis by selecting the best-performing or featuring result.

Descriptive statistics were visualized using the coupled forest plot of sensitivity and specificity, as well as the forest plot of log diagnostic odds ratio, generated by Review Manager (RevMan) version 5.4 (The Cochrane Collaboration, 2020). The pooled diagnostic odds ratio was estimated by meta-analysis using a univariate technique on the per-sample level data. A random effect model was adopted based on the DerSimonian and Laird approach (DerSimonian and Laird, 1986). A bivariate approach that produced pooled sensitivity and specificity was not considered because of the small number of available studies (Gatsonis and Paliwal, 2006) and the fact that the thresholds between studies were different because of the variations in instruments.

The diagnostic odds ratios were displayed using the forest plot, while the confidence intervals of diagnostic accuracy parameters of the Lehmann model (or proportional hazard model) were visualized using the summary receiver operating characteristics curve (SROC) (Holling et al., 2012). A diagnostic odds ratio of 10.00 was considered a good test (Deeks, 2001). Heterogeneity was identified based on qualitative observation of the summary points and plots since I^2^ statistics were inappropriate for meta-analysis of test accuracy (McGrath et al., 2017). Sensitivity analysis and the evaluation of small-study effects were not conducted because of the small number of included studies (Lau et al., 2006). Meta-analysis was performed using R statistical package (Foundation for Statistical Computing, Vienna, Austria) with the “mada” package.

## 3 Study selection

**FIGURE 1:**
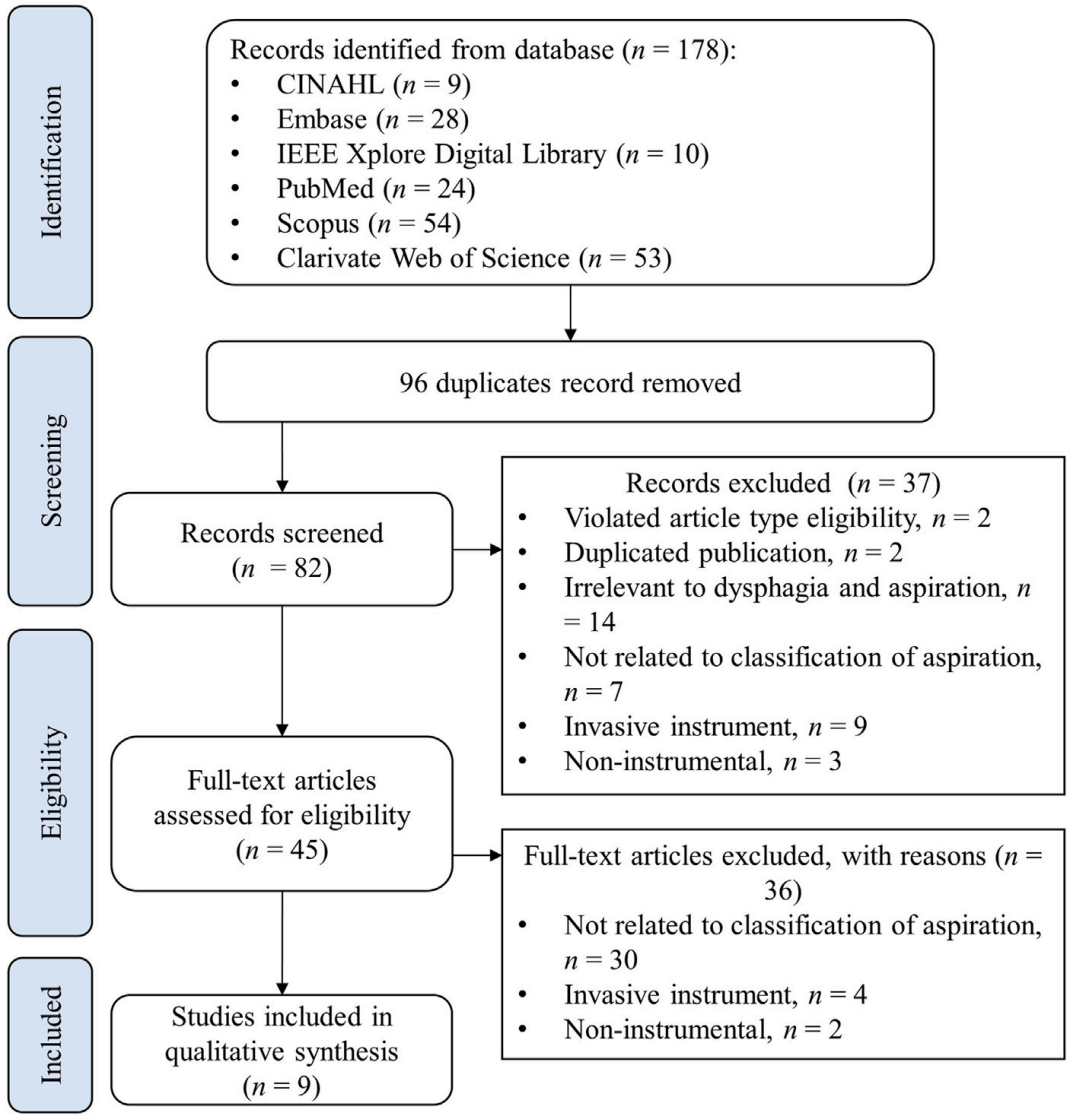
PRISMA flowchart of systematic search and screening.

The PRISMA flowchart shown in Figure 1 illustrates the search and screening process for the review. The initial search identified 178 articles from the six databases, and 96 duplicate articles were removed. Screening on the title and abstracts excluded 37 records, for the following reasons: violation of inclusion criteria of article types, such as reviews and conference abstracts, *n* = 2; duplicate publications (articles published as full conference papers were published again in journals with the same content. In such cases, only publications in journals were retained since they contained full, detailed information), *n* = 2; irrelevant to dysphagia and aspiration, *n* = 14; not related to the classification of aspiration, *n* = *7*; invasive instrument, such as manometry, *n* = 9; non-instrumental, such as questionnaires, *n* = 3. Subsequently, the full-text screening was further performed to exclude 36 articles, of which 30 were not related to the classification of aspiration, 4 involved invasive instruments, and 2 were targeted for non-instrumental screening. In the end, 9 articles were eligible for data synthesis (Lee et al., 2006; Lee et al., 2011; Merey et al., 2012; Sarraf Shirazi et al., 2012; Sejdic et al., 2013; Sarraf Shirazi et al., 2014; Frakking et al., 2022; Park et al., 2022; Shu et al., 2022).

## 4 Study source

There were five studies led by research institutes from Canada (Lee et al., 2006; Lee et al., 2011; Merey et al., 2012; Sarraf Shirazi et al., 2012; Sarraf Shirazi et al., 2014), two from the United States (Sejdic et al., 2013; Shu et al., 2022), one from Australia (Frakking et al., 2022) and one from South Korea (Park et al., 2022). In addition, three of the leading teams were from clinical institutes/departments (Lee et al., 2006; Frakking et al., 2022; Park et al., 2022), while the other eight studies were either from departments of bioengineering/biomedical engineering (Lee et al., 2011) (Merey et al., 2012; Sarraf Shirazi et al., 2012) or departments of electrical and computer engineering (Sejdic et al., 2013; Sarraf Shirazi et al., 2014; Shu et al., 2022). The included studies were published in *Dysphagia* (Frakking et al., 2022), *Journal of Neuroengineering and Rehabilitation* (Lee et al., 2006; Merey et al., 2012), *Artificial Intelligence in Medicine* (Lee et al., 2011), *Medical and Biological Engineering and Computing* (Sarraf Shirazi et al., 2012; Sarraf Shirazi et al., 2014), *IEEE Transaction of Biomedical Engineering* (Sejdic et al., 2013), *IEEE Journal of Biomedical and Health Informatics* (Shu et al., 2022), and *Scientific Reports* (Park et al., 2022) that spanned across the clinical, engineering, and interdisciplinary science fields. It should be noted that the majority of the work has been published or is connected to the same research team.

## 5 Study characteristics

### 5.1 Populations

The eligible studies (*n* = 9) involved a total of 960 participants (427 males, 307 females, and 40 unspecified genders) in their experiments, as shown in Table 2. The sample size of the studies ranged from 10 to 234. The target population was divided into two age groups: children and adults. Three studies (*n* = 187, males = 107, females = 71) involved dysphagic children, of which two recruited children of about 6 years old (Lee et al., 2006; Merey et al., 2012) and the other was about 1 year old (Frakking et al., 2022). Most of them had feeding disorders. Cerebral palsy was among the common pathologies at-risk of aspiration in these studies, in addition to developmental delays. Since Frakking et al. (2022) recruited younger children, they also considered related congenital syndromes, such as Beckwith-Wiedemann syndrome, Cri-du-chat syndrome, and Pierre Robin syndrome. Moreover, Frakking et al. (2022)’s study was the only one to recruit healthy participants as controls among the nine studies.

**TABLE 2:** Participant information.

Article	Population	Sample size	Sex (male: female)	Mean age (years) (SD, range)
Frakking et al. (2022)	Children with feeding disorders	18	11:7	Median age = 10.5 months (range 2–701)
	Typically developing children (healthy control)	23	12:11	Median age = 13 months (range 4–33)
Lee et al. (2006)	Children suspected at risk of aspiration	117	64:53	6.0 (3.9, N/A)
Lee et al. (2011)	Adults suffered from stroke or acquired brain injury	24	20:4	64.8 (18.6, N/A)
Merey et al. (2012)	Children diagnosed with neurological conditions and feeding disorders	29	20:0	6.8 (4.8, N/A)
Park et al. (2022)	Patients with suspected swallowing disorder attributable to brain lesion, excluding neurodegenerative disorders	Mild: 215	135:137	Mild: 65.7 (13.2, N/A)
		Severe: 234		Severe: 72.2 (11.2, N/A)
Sarraf Shirazi et al. (2014)	Dysphagic adult patients with stroke, acquired brain injury, or neurodegenerative disorders	Mild: 39	39:11	60 (20, N/A)
		Severe: 11		
Sarraf Shirazi et al. (2014)	Dysphagia adult patients with stroke, acquired brain injury, or neurodegenerative disorders	21	11:10	Aspirated group: 58.4 (19.1, 23–81)
				No information for non-aspirated group
Sejdic et al. (2013)	Dysphagia patients	40	N/A	N/A
Shu et al. (2022)	Suspected neurogenic dysphagia	189	115: 74	Males: 23–97
				Females: 19–89

N/A, not available; SD, standard deviation.

For studies that recruited adults (*n* = 763, males = 320, females = 236, gender unspecified = 40), the mean ages ranged from 19.0 to 72.2 years. The large age span was due to the different sources of dysphagia that could be divided into neurogenic and neurodegenerative factors. The participants with neurogenic dysphagia could have suffered from stroke or an acquired brain injury, while those with neurodegenerative conditions might be affected by dementia, Parkinson’s disease, or Alzheimer disease. Three studies considered neurogenic dysphagia patients (Lee et al., 2011; Park et al., 2022; Shu et al., 2022), while two considered both neurogenic and neurodegenerative patients (Sarraf Shirazi et al., 2012; Sarraf Shirazi et al., 2014). One study did not specify the potential cause of dysphagia (Sejdic et al., 2013).

Two studies have further considered the sub-classification of dysphagia severity or higher risk of aspiration (Sarraf Shirazi et al., 2014; Park et al., 2022). Park et al. (2022) found that 52.1% (*n* = 234) of the participants were recognized as having severe dysphagia, and 44.9% of these severe cases (*n* = 105) were confirmed to have aspiration pneumonia, pleural effusion, or bronchitis. Besides, Sarraf Shirazi et al. (2014) identified severe dysphagic individuals if they presented more than half or five aspirated swallows in the swallowing assessments.

### 5.2 Index test

#### 5.2.1 Instruments and testing procedures

As shown in Table 3, accelerometric and acoustic techniques were used for the index tests in three (Lee et al., 2006; Lee et al., 2011; Merey et al., 2012) and four studies (Sarraf Shirazi et al., 2012; Sarraf Shirazi et al., 2014; Frakking et al., 2022; Park et al., 2022), respectively, while two studies used both accelerometric and acoustic techniques (Sejdic et al., 2013; Shu et al., 2022). Interestingly, single-axis (Lee et al., 2006), dual-axis (Lee et al., 2011; Merey et al., 2012; Sejdic et al., 2013), and tri-axis accelerometers (Shu et al., 2022) were all reported. A single-axis accelerometer (EMT 25-C, Siemens) was placed inferoanterior to the thyroid notch, receiving signal frequencies from 30 Hz to 20 kHz (Lee et al., 2006). Besides, all three studies that utilized dual-axis accelerometers had aligned them along the anterior-posterior (A-P) and superior-inferior (S-I) directions (Lee et al., 2011; Merey et al., 2012; Sejdic et al., 2013). Merey et al. (2012) processed the dual-axis accelerometric signals by downsampling to 1 kHz (Lee et al., 2008), segmenting through the robust algorithm for pitch tracking (RAPT) (Sejdić et al., 2010a), detrending the low-frequency component using least-square spine approximation (Sejdić et al., 2010b) and then de-noising using the Meyer wavelet transform with soft thresholding. Lee et al. (2011) utilized a similar accelerometer configuration in the A-P and S-I directions but placed the sensor just below the thyroid cartilage. Additionally, the authors incorporated the system with an airflow pressure transducer (PTAF Lite, Grass Technologies) and a nasal cannula (Pro-Flow Cannulas Model 1,259, Glass Technologies) to measure the signal of nasal airflow. They implemented a 5-level discrete wavelet decomposition using Daubechies 5 wavelets and high-passed the signal using a 4^th^ order Butterworth filter with a 1-Hz cutoff frequency.

**TABLE 3:** Instrument configuration and testing protocol.

Article	Modality	Instrument	Configuration	Protocol
Frakking et al. (2022)	Acoustic	Omnidirectional Condenser microphone	Lateral to the cricoid cartilage at C6	Two presentations of puree, lumpy mash, chewable solid, extremely thick, moderately thick, mildly thick, slightly thick and/or thin fluids. Only one swallow on thin fluids per patient was used
Lee et al. (2006)	Accelerometry	Single-axis accelerometer (EMT 25-C)	Inferoanterior to the thyroid notch	Barium-coated boluses of varying consistencies, ranging from thick puree to thin liquid, were fed
Lee et al. (2011)	Accelerometry	Dual-axis accelerometer (ADXL322) for valleculae and pyriform sinuses	Just below the thyroid cartilage	Beginning with a thin liquid 40% weight per volume barium suspension and progressing through nectar- and spoon-thick liquids to solid
	Airflow pressure	Nasal Cannula with pressure transducer (PTAF Lite)	At the nares	
Merey et al. (2012)	Accelerometry	Dual-axis accelerometer (ADXL322)	Level of cricoid cartilage	Barium-coated boluses of varying consistencies, ranging from thick puree to thin liquid, were fed
Park et al. (2022)	Acoustic	Embedded microphone of an iPad	20 cm from patient’s face	Phonate a single syllable for at least 5 s with comfortable pitch and loudness. No swallowing task
Sarraf Shirazi et al. (2014)	Acoustic	Microphone (ECM-77B)	On the suprasternal notch of trachea	Different type of solid/liquid food. Type and order determined by speech pathologist
Sarraf Shirazi et al. (2012)	Acoustic	Microphone (ECM-77B)	On the suprasternal notch of trachea	Different type of solid/liquid food. Type and order determined by speech pathologist
Sejdic et al. (2013)	Accelerometry	Dual-axis accelerometer (ADXL322)	Anterior to the cricoid cartilage	5 mL sips of thin liquid barium
	Acoustic	Lapel microphone	Around the neck	
Shu et al. (2022)	Accelerometry	Tri-axis accelerometer (ADXL327)	Anterior to the cricoid cartilage	Swallowing assessment in clinical routine
	Acoustic	Contact microphone (C411L)	Slightly below the accelerometer anterolateral to larynx	

Sejdic et al. (2013) and Shu et al. (2022) applied dual-axial and tri-axial accelerometers, respectively, and placed them anterior to the cricoid cartilage, in addition to a microphone. Both studies bandpassed the signal from 0.1 Hz to 3 kHz. Besides, Sarraf Shirazi et al. (2014) and Sarraf Shirazi et al. (2012) recorded the sound with a microphone at the suprasternal notch of the trachea at 44.1 kHz and band-passed it at a range between 150 Hz and 5 kHz. The authors attempted to separate the sounds of breathing and swallowing through an aural and visual examination of the time-frequency signal spectrum. On the other hand, while Frakking et al. (2022) made use of an omnidirectional condenser microphone (C417, AKG Acoustics, Vienna, Austria) in the form of a circular O-ring lateral to the cricoid cartilage, Park et al. (2022) recorded the speaking sound using an iPad (with an embedded microphone) placed 20 cm in front of the participants’ faces (Umayahara et al., 2018). The sampling frequency was 44.1 kHz and was band-passed between 20 Hz and 8 kHz. In summary, all studies included the bandwidth of signals between 150 Hz and 3 kHz.

Regarding the protocol for measurement, most of them referred to the standard swallowing assessment procedures that fed food/liquid with different consistencies or thicknesses (Lee et al., 2006; Lee et al., 2011; Merey et al., 2012; Frakking et al., 2022; Shu et al., 2022). Nevertheless, some studies determined the swallowing items by the speech pathologists (Sarraf Shirazi et al., 2012; Sarraf Shirazi et al., 2014). All of them coated/diluted the food/liquid with barium to facilitate the reference test using VFSS. Park et al. (2022)’s study was the only one that did not involve swallowing tasks (neither eating nor drinking). They aimed at the speaking sound and asked the participants to phonate a single syllable for at least 5 s with a comfortable pitch and loudness. The pieces consisted of single vowel phonations that were easy to follow.

#### 5.2.2 Features

As shown in Table 4, predetermined time domain statistical features were commonly adopted in the studies for both accelerometric and acoustic signals, including, mean, variance, standard deviation, median, interquartile range, skewness, and kurtosis (Lee et al., 2006; Lee et al., 2011; Merey et al., 2012; Shu et al., 2022), while some studies implemented variations on the predetermined statistical features, such as normality, significance value of the normality test, and the absolute difference between mean and median (Lee et al., 2006; Lee et al., 2011; Merey et al., 2012). The dispersion ratio was defined as the ratio between the mean absolute deviation (MAD) and the interquartile range (IQR) (Lee et al., 2006), while the feature of stationarity reflected that the mean and variance of the signal did not change with time and was calculated using the reverse arrangement test (Bendat and Piersol, 2011). Energy, entropy rate, and Lempel-Ziv complexity were also considered in the studies. The maximum hyolaryngeal excursion was estimated by double integrating the accelerometer signal (Lee et al., 2011; Merey et al., 2012). Jitter and shimmer features were commonly accounted for in acoustic signals, including a series of features on the perturbation quotient (Park et al., 2022). Park et al. (2022) concatenated those features with and without clinical data of the participants.

**TABLE 4:** Summary of extracted features from accelerometric and acoustic signals for aspiration risks classification.

Modality	Domain	Feature	Reference
Accelerometry	Time	Statistical features (mean, median, variance, skewness, kurtosis, Interquartile Range), Absolute difference between mean and median Stationary, normality, dispersion ratio Significance level of normality, Maximum hyolaryngeal excursion, proportion of signal corresponding to maximum hyolaryngeal excursion Zero-crossing Energy, entropy rate, Lempel-Ziv complexity Linear prediction coefficient	Lee et al. (2006)
			Lee et al. (2011)
			Merey et al. (2012)
			Shu et al. (2022)
	Frequency	Peak frequency, centroid frequency Band width Peak Fast Fourier Transform magnitude, frequency at spectral peak Frequency corresponding to max spectral density over time of the short-time Fourier transform Difference between frequency corresponding to 75% and 25% of max spectral density at time corresponding to max frequency Statistical features of power spectrum	Lee et al. (2011)
			Merey et al. (2012)
			Shu et al. (2022)
	Time-frequency	Wavelet entropy and energy Wavelet packet coefficient Relative energy and entropy for wavelet decomposition	Lee et al. (2011)
			Sejdic et al. (2013)
			Shu et al. (2022)
Acoustics	Time	Statistical features (standard deviation, skewness, kurtosis) Entropy rate, Lempel-Ziv complexity Phase-space thresholding, Normalized energy of the 3^rd^ quartile of average power Jitter and shimmer features	Sarraf Shirazi et al. (2012)
			Sarraf Shirazi et al. (2014)
			Park et al. (2022)
			Shu et al. (2022)
	Frequency	Peak frequency, centroid frequency Band width	Frakking et al. (2022)
			Shu et al. (2022)
	Time-frequency	Wavelet Entropy Wavelet packet coefficient	Sejdic et al. (2013)
			Shu et al. (2022)

Sarraf Shirazi et al. (2014) proposed using features inspired by the phase-space thresholding technique that originated from acoustical doppler velocimetry (Cea et al., 2007). In brief, the acoustic signals were plotted against the first and second derivatives and fitted with an ellipsoid. The summed distance between the points outside the ellipsoid and the ellipsoid center were calculated and normalized to the total energy (in the time domain) to serve as the feature. Another paper from the team (Sarraf Shirazi et al., 2012) reported another feature targeting the average power values. They calculated the sum of the squared values for those greater than the third quartile and normalized it to the sum of all squared values.

Typical frequency domain signal features included peak frequency, centroid frequency, band width, peak Fast Fourier Transform (FFT) magnitude, and frequency at the spectral peak (Lee et al., 2011; Merey et al., 2012; Shu et al., 2022). Besides, Merey et al. (2012) inspected the frequency features on the spectral density spectrogram, including that maximum, the difference between 75% and 25% of the maximum spectral density, in addition to 20 more features derived by the summation of power spectral density values.

Features related to wavelets and wavelet decomposition were related to the time-frequency domain, which helped capture nonstationary nature of signals (Chau et al., 2005). Besides the energy and entropy of the wavelets, Sejdic et al. (2013) extracted the wavelet packet coefficient from the discrete wavelet transform series. Particularly, the authors compared and evaluated the combinations of different wavelets (Coiflet and Meyer) and time-frequency domain features of wavelets (log-energy and entropy) on the A-P and S-I axes signals of the accelerometers (Sejdic et al., 2013).

#### 5.2.3 Modeling (classifiers)

Six studies conducted the classification at the per-sample level (i.e., classifying risky swallowing samples), while two studies conducted the classification at the per-individual level (i.e., classifying risky individuals). One study accounted for both per-sample and per-individual levels. SVM was among the most popular and promising classifiers in the review (Merey et al., 2012; Sarraf Shirazi et al., 2014; Frakking et al., 2022; Shu et al., 2022), as shown in Table 5. It is a supervised machine learning model that separates data into categories (classification) by finding the best hyperplane in a n-dimensional space (where n is the number of features). Frakking et al. (2022) trained the SVM with a 50:50 training-to-testing ratio and subsequently performed hyperparameter tuning using grid search through 5-fold cross-validation; Sarraf Shirazi et al. (2014) distinguished individuals with severe aspiration using SVM, which input a phase-space representation of breathing sound. Literature has compared the performance of SVM with other statistical models/machine learning models. For example; Shu et al. (2022) compared SVM with k-means, Naive Bayes, and an artificial neural network (ANN). Park et al. (2022) compared SVM with logistic regression, decision tree, random forest, Gaussian mixture model, and extreme gradient boosting (XGBoost). In fact, Park et al. (2022) adopted a two-step classification approach. First, they identified individuals with severe dysphagia and those with mild or minimal cases. Then, for those severe dysphagia cases, they identified whether they had a risk of respiratory complications (not included in the tables). Hyperparameters were not tuned but assigned default values.

**TABLE 5:** Modeling and model training strategy.

Article	Binary classifier	Swallow sample (aspirated/unsafe vs. normal)	Reference test	Training strategy
Frakking et al. (2022)	SVM	18 vs. 106	VFSS	50:50 training-to-testing ratio, 5-fold CV for hyperparameter tuning
Lee et al. (2006)	RBF	94 v. 100	VFSS	10-fold CV
Lee et al. (2011)	3 channels (airway invasion, valleculae clearance and pyriform sinuses bolus clearance) on 9 classifiers (LDA Euclidean, LDA Mahalanobis, NN (10, 20, 30 HUs), PNN, and KNN (K = 11, 21, 31)	Airway invasion: 39 vs. 265 Valleculae BC: 64 vs. 61 Pyriform sinuses BC: 25 vs. 129	VFSS	10-fold CV
Merey et al. (2012)	LDA w/Euclidean, LDA w/Mahalanobis, SVM linear, SVM RBF, SVM RBF + B2 optimizer	94 vs. 544	VFSS	8-fold CV, bootstrapping to balance class
Park et al. (2022)	Logistic Regression, Decision Tree, Random Forest, SVM, GMM, XGBoost	N/A (per-patient)	VFSS and spirometry	-
Sarraf Shirazi et al. (2014)	SVM	N/A (per-patient)	VFSS or FEES	Leave-one-out
Sarraf Shirazi et al. (2012)	Minimum distance classifier	N/A (per-patient)	VFSS or FEES	Leave-one-out
	Fuzzy k-means clustering	32 vs. 128		
Sejdic et al. (2013)	Bayes	-	VFSS	Leave-one-out
Shu et al. (2022)	SVM, k-means, Naive Bayes, ANN	378 vs. 1701	VFSS	10-fold CV

ANN, artificial neural network; BC, bolus clearance; CV, cross-validation; FEES, fiberoptic endoscopic evaluation of swallowing; GMM, gaussian mixture model; HU, hidden units; KNN, k-nearest-neighbor; LDA, linear discriminant analysis; N/A, not applicable; NN, feed-forward non-linear classifier; PNN, probabilistic neural network; RBF, radial basis function; SVM, support vector machine; XGBoost, Extreme gradient boosting; VFSS, videofluoroscopic swallowing study; w/: with.

A similar two-step classification was adopted by Sarraf Shirazi et al. (2014). They classified the individuals into aspirated and non-aspirated groups. Then, they classified the risky swallows as part of the aspirated group. The former was facilitated by a minimal distance classifier (without addressing the kind of minimal distance classifier) on the normalized energy feature of the third quartile, while the latter was entertained by the unsupervised model, fuzzy k-means clustering. Hyperparameter tuning was conducted by repeating the distance-based probability distribution until the cost function reached a local minimum.

While Lee et al. (2006) evaluated the performance of a radial basis function (RBF) classifier with different combinations of features, their later work (Lee et al., 2011) tested four classifiers with a total of nine paradigms, including linear discriminant analysis (LDA) using Euclidean and Mahalanobis distance measures, feed-forward non-linear (NN) classifiers with 10, 20, and 30 hidden units, a probabilistic neural network (PNN) and K-nearest-neighbor (KNN) with 11, 21, and 31 neighbors. The data were resampled to generate 10,000 samples per class. Regularization was followed by an early stop on the cross-validation to prevent overfitting. Similarly, Merey et al. (2012) also applied the LDA approach but reduced the dimensionality of features by principal component analysis (PCA). Additionally, Merey et al. (2012) evaluated SVM with a linear kernel, an RBF kernel, and an RBF kernel with a B2 optimizer (Jolliffe, 1972). Besides, Sejdic et al. (2013) applied Bayes classifiers and compared different wavelets and their spectrum features (log-energy or entropy) of the A-P and S-I components of the dual-axis accelerometer.

### 5.3 Reference test

The VFSS served as the reference test for all papers, while some also considered the FEES (Sarraf Shirazi et al., 2012; Sarraf Shirazi et al., 2014) and spirometry (Park et al., 2022) (Table 5). The presence or risk of aspirating swallows or aspirating individuals was determined by physicians examining the VFSS/FEES, especially speech pathologists. Spirometry through peak cough flow (Kulnik et al., 2016) was used to evaluate the risk of respiratory complications (Park et al., 2022). The penetration-aspiration scale was commonly used to help physicians make diagnoses more objectively (Rosenbek et al., 1996), despite the fact that there was a variation on how to use the scale. The total score for the scale was eight, in which scores above six represented entries of bolus below the level of vocal cords and were regarded as aspiration swallows. Both Sejdic et al. (2013) and Shu et al. (2022) set a threshold above three for “unsafe” swallows. Moreover, Lee et al. (2011) rated the swallows in three domains: airway invasion, bolus clearance at valleculae, and bolus clearance at pyriform sinuses, which were rated by the 4-point depth of airway invasion scale and the 4-point bolus clearance scale, respectively. Only cases rated at levels 0 (safe) and 3 (materials entering the airway/substantial residual material filling or overflowing) were investigated in the study. Besides, Merey et al. (2012) used a 3-point swallowing rating (0: materials do not enter the airway; 1: materials enter the airway but do not pass below the vocal folds; 2: materials enter the airway and pass below the vocal folds) and only selected participants that rated zero and two in their study.

### 5.4 Outcome and performance evaluation

**FIGURE 2:**
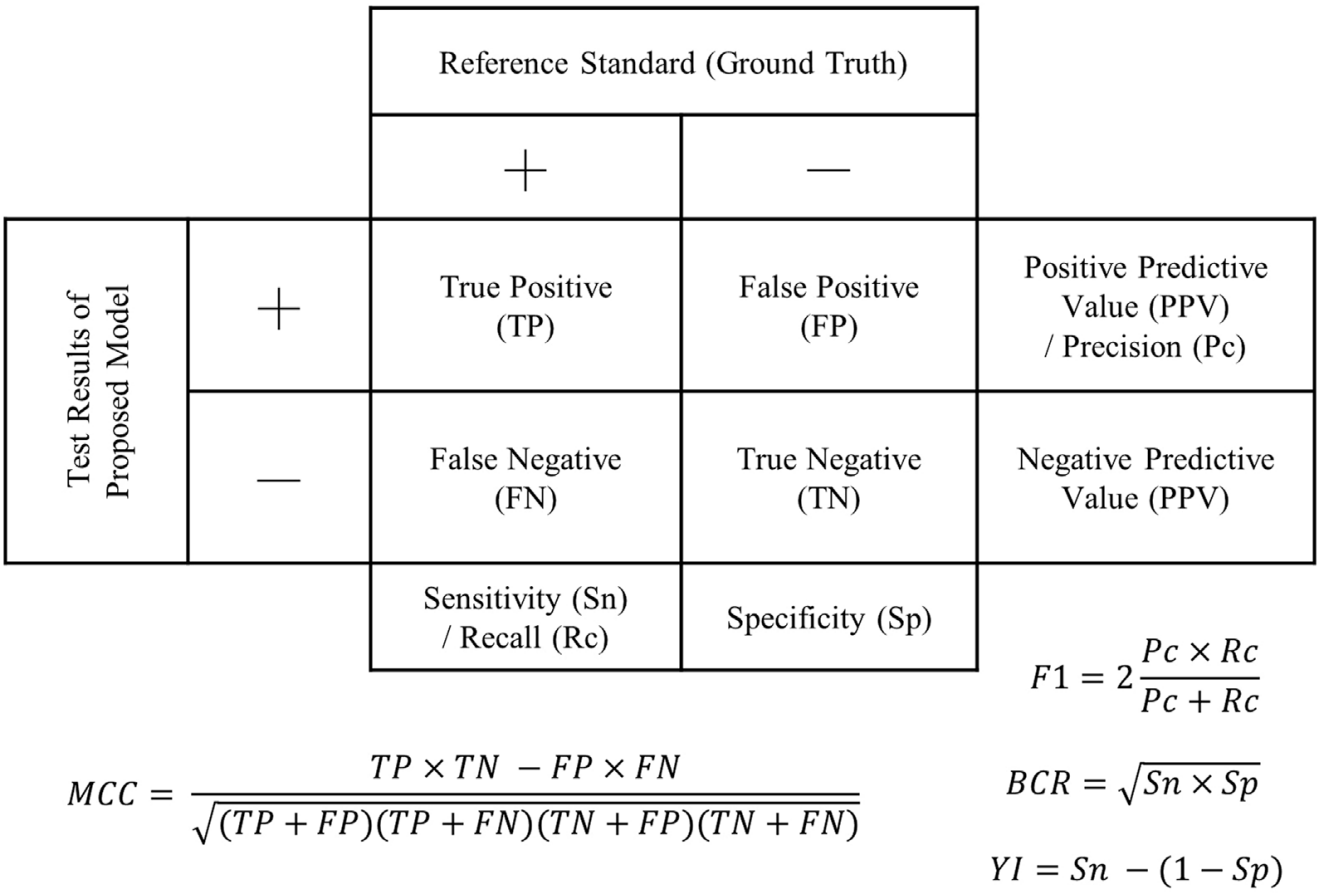
Confusion matrix (2 × 2 contingency table) demonstrating outcome measures for accuracy evaluation. F1: F1-score; BCR, balanced classification rate; MCC, Matthew’s correlation coefficient; Y1, Youden’s index.

Accuracy, sensitivity, and specificity were the standard outcome measures used to evaluate diagnostic/screening accuracy and were derived from the confusion matrix (or 2 × 2 contingency table) (Figure 2). Accuracy is the ratio of correct tests to the total number of tests. Sensitivity shows the proportion of positive diagnoses from the index test that are also detected as positive by the reference test, while specificity indicates the proportion of negative diagnoses from the index test that are also detected as negative by the reference test. Three studies reported the F1-score. The F1-score quantifies the balance between precision (PPV) and recall (or sensitivity) by taking the harmonic mean, which partially accounts for the imbalanced class problem but does not take into account the cost of misclassifying the minor class. Shu et al. (2022) reported the Matthews Correlation Coefficient (MCC), which ranges from −1 (complete disagreement) to +1 (perfect agreement), with 0 indicating random predictions. Besides, AUC manifests the discrimination capability of a binary classifier by plotting the sensitivity and specificity at different classification thresholds.

Highly imbalanced classes are a prevalent issue in healthcare and medicine (Jothi and Husain, 2015; Mao et al., 2022; Mao et al., 2023) since it is natural to have fewer positive than negative cases (i.e., non-healthy cases are often underrepresented), which was also reflected in our review (Table 4). It should be noted that some studies defined “adjusted accuracy” by taking a simple average of sensitivity and specificity and claimed that the parameter could resolve the imbalanced class issue, with which we disagreed. In fact, resampling (Lee et al., 2011), bootstrapping (Merey et al., 2012), data augmentation (Shu et al., 2022), and Mahalanobis distance measures were applied to accommodate the imbalanced class problem. There were also other oversampling techniques (Santos et al., 2018), such as the Synthetic Majority Oversampling Technique (SMOTE) and the Adaptive Synthetic Sampling Approach (ADASYN).

To calculate the accuracy performance, testing data that are independent of the data for model training (or fitting) are essential to ensure that the model can generalize well to new data (i.e., to prevent overfitting) (Poldrack et al., 2020). Frakking et al. (2022) used half of the data for training and half for testing, even though the authors utilized the cross-validation technique for hyperparameter tuning. Nevertheless, one study did not specify whether they had implemented an independent testing set (Sarraf Shirazi et al., 2012). In fact, cross-validation is a technique to facilitate independent testing with a relatively small sample size. It involves verifying the accuracy of the model by dividing subsets (folds) of training and testing data and calculating their average performance. Our review found that existing studies applied 8-fold (Merey et al., 2012), 10-fold (Lee et al., 2006; Lee et al., 2011; Shu et al., 2022), and leave-one-out (Sarraf Shirazi et al., 2012; Sejdic et al., 2013; Sarraf Shirazi et al., 2014) cross-validation.

Table 6 shows the key findings of the studies. Since some studies presented lengthy results of different combinations of features/hyperparameters, we only included the results of the best-performing combination for Lee et al. (2006), Lee et al. (2011), and Sejdic et al. (2013). In addition, Shu et al. (2022) evaluated different data augmentation strategies, and we presented that with AC-GAN (auxiliary classifier Wasserstein generative adversarial network), which was the targeted innovation of the paper. Moreover, we presented outcomes for Park et al. (2022) that made use of the acoustic signal data only (i.e., did not present the results for acoustic plus clinical data).

**TABLE 6:** Outcome metrics and test performance.

Article	Classifier	Test performance outcome metrics						
		Acc	Sn/Rc	Sp	PPV/Pc	NPV	AUC	Others
Frakking et al. (2022)	SVM	98	89	100	100	100	-	F1: 0.94
Lee et al. (2006) ^(^ ^a^ ^)^	RBF	82.1	74.7	87.8	-	-	-	Adj. accuracy: 81.3
Lee et al. (2011) ^(^ ^a^ ^)^	Airway invasion: LDA Euclidean	-	100	49.4	-	-	-	Adj. accuracy: 74.7
	Valleculae BC: LDA Mahalanobis	-	75.5	91.9	-	-	-	Adj. accuracy: 83.7
	Pyriform sinuses BC: LDA w/Mahalanobis	-	81.7	86.8	-	-	-	Adj. accuracy: 84.2
Merey et al. (2012)	LDA w/Euclidean	62.8	50.7	74.9	-	-	-	-
	LDA w/Mahalanobis	60.6	69.8	51.4	-	-	-	-
	SVM linear	62.0	51.5	72.4	-	-	-	-
	SVM RBF	80.6	80.0	81.2	-	-	-	-
	SVM RBF + B2 optimizer	86.9	89.6	92.2	-	-	-	-
Park et al. (2022) ^(^ ^b^ ^)^	Logistic Regression	68.2	65.7	70.7	69.3	67.8	0.69	F1: 0.67
	Decision Tree	69.0	62.0	76.0	73.3	66.6	0.70	F1: 0.67
	Random Forest	73.7	70.7	76.7	75.7	72.5	0.78	F1: 0.73
	SVM	69.7	71.0	68.3	69.4	70.2	0.68	F1: 0.70
	GMM	66.2	64.7	67.7	66.3	67.5	0.64	F1: 0.64
	XGBoost	74.8	72.7	77.0	76.8	74.8	0.78	F1: 0.74
Sarraf Shirazi et al. (2014)	SVM	86.0	91.0	84.0	-	-	-	-
Sarraf Shirazi et al. (2012)	Classify population: min distance classifier	90.0	-	-	-	-	-	-
	Classify swallow: fuzzy k-means	86.4	86.4	86.4	61.5	96.2	-	-
Sejdic et al. (2013) ^(^ ^a^ ^)^	Bayes	94.6	92.5	95.6	-	-	-	-
Shu et al. (2022)	Naïve Bayes w/AC-GAN	66.38	39.03	74.6	-	-	-	F1: 22.02
								MCC: 0.0324
	K-means w/AC-GAN	72.94	12.40	86.41	-	-	-	F1: 13.24
								MCC: −0.0009
	SVM w/AC-GAN	75.02	21.71	86.84	-	-	-	F1: 22.83
								MCC: 0.0938
	ANN w/AC-GAN	71.39	32.84	79.78	-	-	-	F1: 28.75
								MCC: 0.1171

Classifier column: AC-GAN, auxiliary classifier Wasserstein generative adversarial network; BC, bolus clearance; GMM, gaussian mixture model; LDA, linear discriminant analysis; RBF, radial basis function; SVM, support vector machine; XGBoost, Extreme gradient boosting; w/, with. Outcome metrics column: Acc, accuracy; AUC, area under receiver-operating curve; NPV, negative predictive value; Pc, precision; PPV, positive predictive value; Rc, recall; Sn: sensitivity; Sp, spec-ificity.

^a^ Classifiers with feature combination of the best accuracy/adjusted accuracy are shown in this table.

^b^ Performance for classifying mild/severe dysphagia or aspirated using model trained by acoustics only (without clinical data) is shown in this table.

We found three studies with excellent accuracy (≥90%) (Sarraf Shirazi et al., 2012; Sejdic et al., 2013; Frakking et al., 2022), while four studies had an accuracy or adjusted accuracy between 80% and 90% (Lee et al., 2006; Lee et al., 2011; Merey et al., 2012; Sarraf Shirazi et al., 2014). Two studies demonstrated an accuracy <80%. Frakking et al. (2022) and Sejdic et al. (2013) achieved accuracy of 98% and 94.6% using SVM and Bayes, respectively. In addition, the latter picked the log-energy features and considered the Coiflet-5 and Coiflet-3 wavelets for A-P and S-I accelerometry, respectively. Sarraf Shirazi et al. (2012) could identify unsafe swallows with 86.4% accuracy. While Lee et al. (2006) compared 31 feature combinations, the best yield was using the dispersion ratio, energy, and normality at 82.1% accuracy. The same team measured the accelerometry of pyriform sinuses using LDA Mahalanobis produced a sensitivity and specificity of more than 80%. Merey et al. (2012) performed a bit better, with an accuracy of 86.9% using SVM with an RBF kernel and B2 optimizer (for feature reduction). Using the proposed AC-GAN, the classification performance for SVM was 75.0%, reported by Shu et al. (2022). Nonetheless, classification performance for other GAN models seemed to be better than the proposed one. Park et al. (2022) classified the acoustic signal using XGBoost, which produced an accuracy of 74.8%, yet the performance was better than the model using both the acoustic signal and clinical data.

## 6 Study quality (risk of bias and applicability)

**FIGURE 3:**
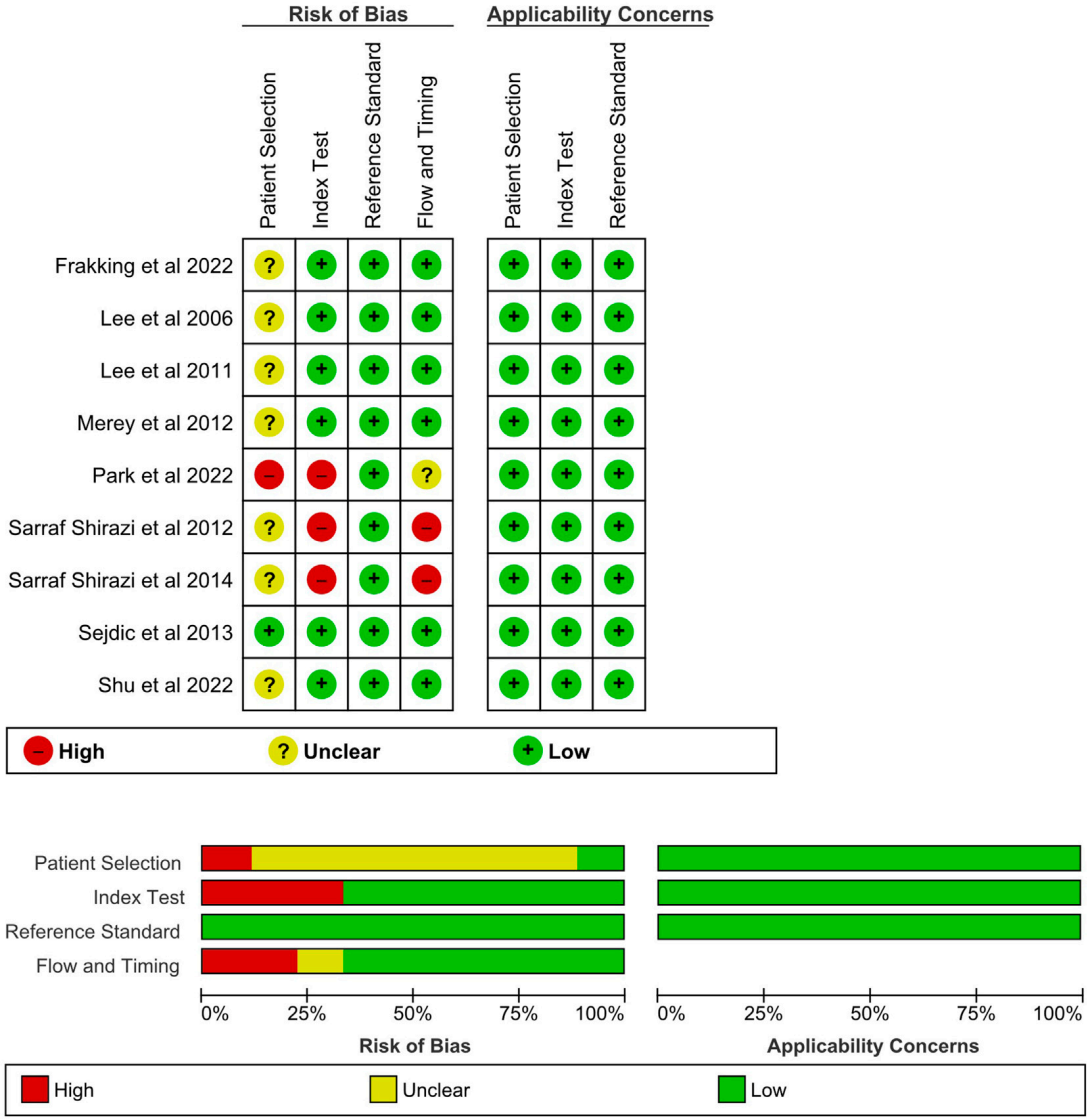
Study quality of the reviewed studies assessed by QUADAS-2.

Out of the seven items, the average point of the studies was 5.44, with a standard deviation of 1.13 (Figure 3). All items under applicability concerns were scored since all studies provided physician diagnosis and benchmarking instrument data to justify the patients and/or events. Nearly all papers lost points on the patient selection domain without clarifying whether the participants were recruited consecutively or by random sampling. All except one study conducted the index test and reference test simultaneously, while some studies lost points for not using the same reference standard. Besides, a risk of bias was also found for studies that excluded patients because they could not complete the test or discarded data with problems.

## 7 Meta-analysis

Among the nine included studies, five were further processed for meta-analysis. One study was discarded due to the lack of sample count information (Sejdic et al., 2013). Two studies were discarded because they were not classifying risky swallows (i.e., not per-sample level) but individuals at risk of aspiration (i.e., per-individual level) (Sarraf Shirazi et al., 2014; Park et al., 2022). We eliminated one study because the leave-one-out validation cannot be used to estimate the sample counts (Sarraf Shirazi et al., 2012).

**FIGURE 4:**
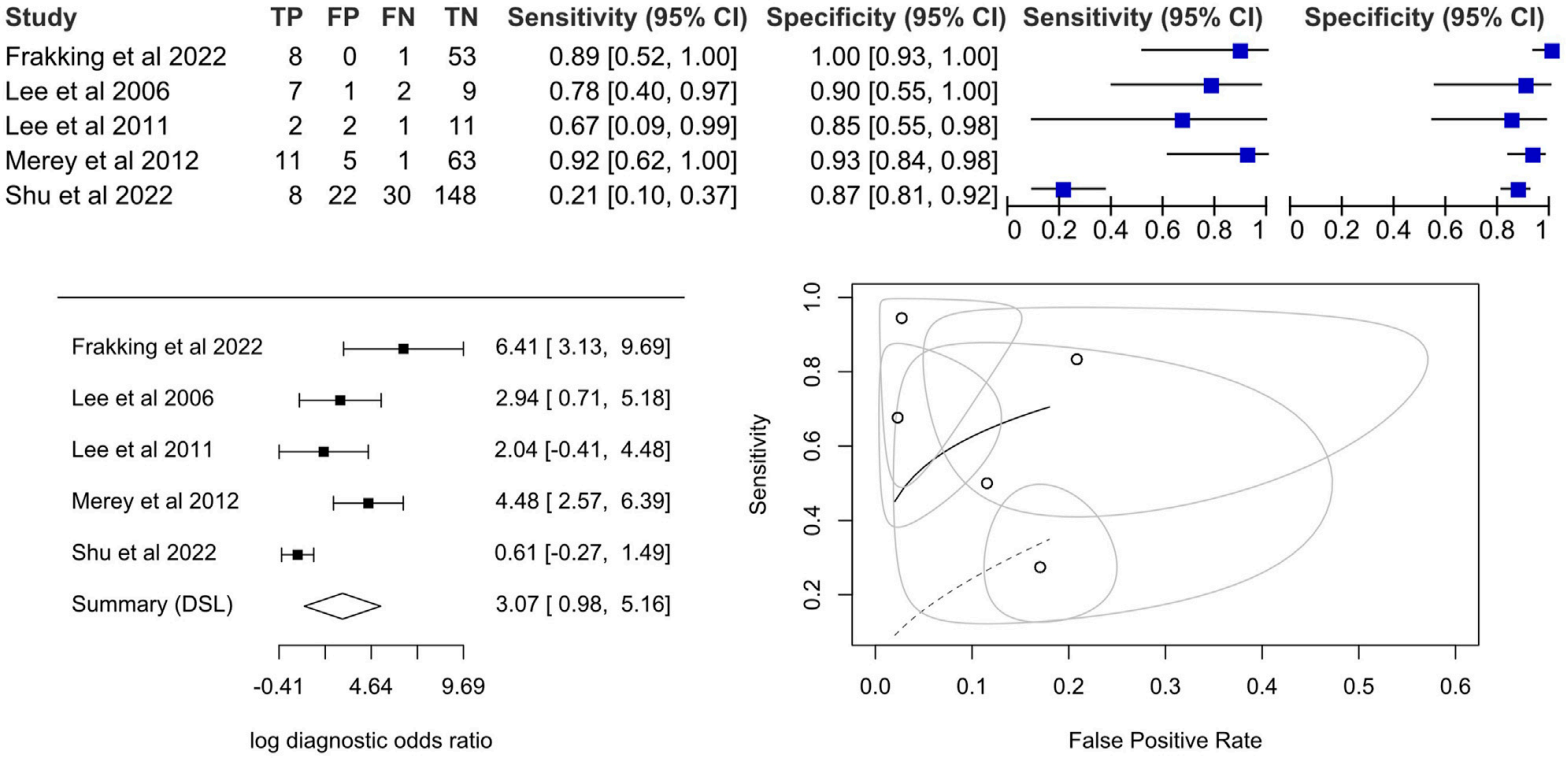
Coupled forest plot on the sensitivity and specificity, forest plot for a univariate random effects meta-analysis using diagnostic odds ratio and summary plot of proportional hazard model (Lee et al., 2006; Lee et al., 2011; Merey et al., 2012; Frakking et al., 2022; Shu et al., 2022).

The pooled diagnostic odds ratio was 21.5 (95%CI, 2.7–173.6), which was higher than the cut-off of 10.00 (Deeks, 2001) but not significant. The coupled forest plot and the forest plot of the log diagnostic odds ratio (Figure 4) demonstrated that there were high standard errors within studies that might be due to small sample sizes, in addition to variations between studies. Sensitivity could be as low as 21% (95%CI, 10%–37%) while specificity could be as high as 100% (95%CI, 93%–100%), in individual studies. By observing the SROC plot (Figure 4), it could be seen that the study-level data points dispersed over the ROC space, far away from the summary line, and with a large confidence region, which demonstrated substantial heterogeneity. In view of this, we decided not to conclude the meta-analysis result.

## 8 Discussion

The significance of this study lies in its ability to summarize the accuracy performance and assessment techniques used in computer-aided screening for dysphagia risks using biophysical sensors. While overall accuracy has often been reported as an indicator of model performance, sensitivity is a more clinically important attribute for screening tools to identify those at greater risk (Wirth et al., 2016). Our review demonstrated that the current systems in our included studies were insufficient, with only two studies (with their best optimized models) demonstrating a sensitivity of more than 90%. Interesting, more information seemed not to produce better results (e.g., concatenating clinical history data (Park et al., 2022) and integrating tri-axial accelerometer and acoustic signals (Shu et al., 2022)). There is a need to improve the generalizability of the system with a larger dataset, and optimize the signal processing, segmentation, feature extraction, classifier, and their combinations to improve the accuracy performance.

Clinical heterogeneity could be sought from gender and age-group, the source of dysphagia/aspirations, and the assessment protocols. Gender could be a significant confounder in this scenario, with the Adam’s apple and deeper voice in adult males apparently influencing the throat biomotion and acoustic signals (So et al., 2023). We found no study that submitted gender as an input feature. On the other hand, dysphagic aspiration could be sourced from different pathophysiologies (Wirth et al., 2016). For example, age-related dysphagia (i.e., presbyphagia) demonstrated reduced tongue pressure and delayed triggering of swallow reflection (Rofes et al., 2010), while dementia was characterized by a compulsive eating pattern and a large bolus size (Langmore et al., 2007). Besides, post-stroke patients experienced decreased activation of swallowing because of the lesion of motor neurons (Teismann et al., 2011). These differences in abnormalities might render different signal patterns of aspiration risks, which could be the reason for the large standard error of the study and the high heterogeneity between studies. Lastly, while studies followed a “routine swallowing assessment” protocol by taking different constituencies and thicknesses of food/liquid, the procedure details were vague, and we are uncertain whether all or some swallow trials were selected for the development of computer-aided screening. In addition, only one study accounted for the non-swallowing task. A previous review commented that protocol heterogeneity might hinder the translational potential of wearable technology on swallowing assessment (So et al., 2023) and that a unified framework was necessary to account for both swallowing and non-swallowing activities (Lim et al., 2023).

Besides methodological heterogeneity in terms of instruments, feature extraction, and modeling, some technical issues might exist. The performances between studies were very extreme, ranging from 21.7% to 100%. It is skeptical when accuracy falls below 50%, which is worse than random guessing. While achieving good accuracy is desirable, obtaining perfect accuracy (i.e., 100%) from predictive models is not possible in practice, since they are designed to approximate underlying constitutive relationships by fitting with the stochastic nature of data and algorithms (i.e., a simplified construct related to a part of reality). Skeptical performances could be due to underfitting (datasets too small), overfitting, imbalance classes, misspecification of hyperparameters, and regularization (Boulesteix and Schmid, 2014; Lever et al., 2016; Kaur et al., 2019; Nichols et al., 2019; Weerts et al., 2020). While several studies did not conduct hyperparameter tuning, cross-validation techniques were often used and believed to relieve overfitting but might be prone to data snooping or peeking (Bzdok et al., 2017) and generate biased estimates, especially with small sample size (Vabalas et al., 2019). Five studies recruited fewer than 50 participants (i.e., independent samples). Data samples were subsequently pooled through repeated measurements and data augmentation techniques. Small datasets may produce strongly spurious patterns. As a rule of thumb, 50 samples or 10 samples per feature (Pedregosa et al., 2011; Riley et al., 2020; Scikit-learn developers, 2023) are minimally needed to fit predictive or machine learning models. In practice, more samples are required with higher data dimensionality and the complexity of learning algorithms (Bzdok et al., 2017). Lastly, it is important to scrutinize flaws in the data and models.

There were some limitations in this study. Only English studies were included in this study, which might lead to language bias. Besides, the number of included studies was relatively small, especially since several of them were from the same research team. Their findings might not be independent. Sterne et al. (2011) advised that a minimum of ten studies be reviewed to achieve sufficient power to assess small-study effects as a rule of thumb. On the other hand, we anticipated that flexible electronics, or soft sensors (Jung et al., 2020; Chen et al., 2021c; Gao et al., 2021; Guan et al., 2021), would be included in this review in the first place, but in vain because most of them were still on the research bench from clinical studies. Moreover, we did not conduct an in-depth data synthesis on the signal processing techniques, which warranted another technical review. For the meta-analysis, with the small dataset and unclear risk of consecutive/random sampling, it is likely that the pooled estimates of the meta-analysis lack generalizability and could be misleading. While we endeavored to provide an overall estimate of the area, we discovered that studies had unique methodological characteristics and major differences in the sets of parameters/thresholds. It might not be appropriate to summarize their test performance using meta-analysis. Subgroup analyses were not conducted on different instruments (accelerometers and microphones) and populations (older adults and children) because of the small number of available studies. Besides, studies using a cross-validation approach that did not have well-defined counts of testing sets approximated the confusion matrix based on the averaged results of cross-validation folds and the fold proportion, which might not be viewed as a pertinent method in meta-analysis. With increasing research using machine learning models for diagnostic or screening purposes, there is a need for developing a new meta-analytic approach targeting cross-validation and data resampling.

More effort is yet necessary to improve the accuracy performance of the computer-aided screening systems to identify aspiration risks, in addition to tests on larger sample sizes to ensure generalizability. A similar conclusion has been reached by another review that targeted on systems classifying swallowing and non-swallowing (e.g., speaking, yawning) events (So et al., 2023). Deep learning models were not implemented, which might be due to an insufficient dataset or a lack of a pretrained model. One study utilized the GAN approach to “generate” more data. Future work may consider improving the robustness and establishing protocols for pragmatic exploitation and implementation. Compliance could be a problem, especially with sensors that have to be stuck on the neck (e.g., accelerometers). Patient-centered designs and feasibility studies could be necessary to promote acceptance among patients and caregivers, especially those with dementia (Merilahti et al., 2009; Gold et al., 2018). Furthermore, these biophysical sensors could be incorporated and improve swallowing therapy through gaming, virtual reality, and biofeedback (Li et al., 2016; Mizoguchi et al., 2021; So et al., 2022).

## Data Availability

The original contributions presented in the study are included in the article/supplementary material, further inquiries can be directed to the corresponding authors.
